# A Randomized, Factorial Phase II Study to Determine the Optimal Dosing Regimen for ^68^Ga-Satoreotide Trizoxetan as an Imaging Agent in Patients with Gastroenteropancreatic Neuroendocrine Tumors

**DOI:** 10.2967/jnumed.121.261936

**Published:** 2022-03

**Authors:** Irene Virgolini, Shadfar Bahri, Andreas Kjaer, Henning Grønbæk, Peter Iversen, Esben A. Carlsen, Mathias Loft, Ulrich Knigge, Johanna Maffey-Steffan, Christine Powell, Colin G. Miller, Thomas Rohban, Sandy McEwan, Johannes Czernin

**Affiliations:** 1 Department of Nuclear Medicine, University of Innsbruck, Innsbruck, Austria;; 2 Ahmanson Translational Theranostics Division, Department of Molecular and Medical Pharmacology, David Geffen School of Medicine, UCLA, Los Angeles, California;; 3 Department of Clinical Physiology, Nuclear Medicine and PET and Cluster for Molecular Imaging, Department of Biomedical Sciences, Rigshospitalet, University of Copenhagen, Copenhagen, Denmark;; 4 Department of Hepatology and Gastroenterology, Aarhus University Hospital, Aarhus, Denmark;; 5 Department of Nuclear Medicine and PET Center, Aarhus University Hospital, Aarhus, Denmark;; 6 Department of Endocrinology PE and Department of Surgery C, Rigshospitalet, University of Copenhagen, Copenhagen, Denmark;; 7 Ipsen Bioscience, Cambridge, Massachusetts;; 8 Bracken Group for Ipsen Bioscience, Newtown, Pennsylvania; and; 9 Partner 4 Health for Ipsen Bioscience, Paris, France

**Keywords:** ^68^Ga-satoreotide trizoxetan, neuroendocrine tumors, somatostatin receptor antagonist, diagnostic imaging, optimal dose

## Abstract

^68^Ga-satoreotide trizoxetan is a novel somatostatin receptor antagonist associated with high sensitivity and reproducibility in neuroendocrine tumor (NET) detection and localization. However, the optimal peptide mass and radioactivity ranges for ^68^Ga-satoreotide trizoxetan have not yet been established. We therefore aimed to determine its optimal dosing regimen in patients with metastatic gastroenteropancreatic NETs in a prospective, randomized, 2 × 3 factorial, multicenter phase II study. **Methods:** Patients received ^68^Ga-satoreotide trizoxetan at a peptide mass of 5–20 µg on day 1 of the study and of 30–45 µg on days 16–22, at 1 of 3 ^68^Ga radioactivity ranges (40–80, 100–140, or 160–200 MBq). Whole-body PET/CT imaging was performed 50–70 min after each injection. The primary endpoint was the detection rate of NET lesions imaged by ^68^Ga-satoreotide trizoxetan relative to contrast-enhanced CT (for each of the 6 peptide mass and radioactivity range combinations). **Results:** Twenty-four patients were evaluated in the per-protocol analysis. The median number of lesions detected by ^68^Ga-satoreotide trizoxetan PET/CT or PET alone was at least twice as high as the number detected by contrast-enhanced CT across the 6 studied peptide mass and radioactivity range combinations. There were no differences between the 2 peptide mass ranges or between the 3 radioactivity ranges in the number of identified lesions. However, a trend toward a lower relative lesion count was noted in the liver for the 40- to 80-MBq range. No relationship was observed between the radioactivity range per patient’s body weight (MBq/kg) and the number of lesions detected by ^68^Ga-satoreotide trizoxetan. The median diagnostic sensitivity of ^68^Ga-satoreotide trizoxetan PET/CT, based on the number of lesions per patient, ranged from 85% to 87% across the different peptide mass and radioactivity ranges. Almost all reported adverse events were mild and self-limiting. **Conclusion:** A radioactivity of 100–200 MBq with a peptide mass of up to 50 µg was confirmed as the optimal dosing regimen for ^68^Ga-satoreotide trizoxetan to be used in future phase III studies.

## Visual Abstract

**Figure:**
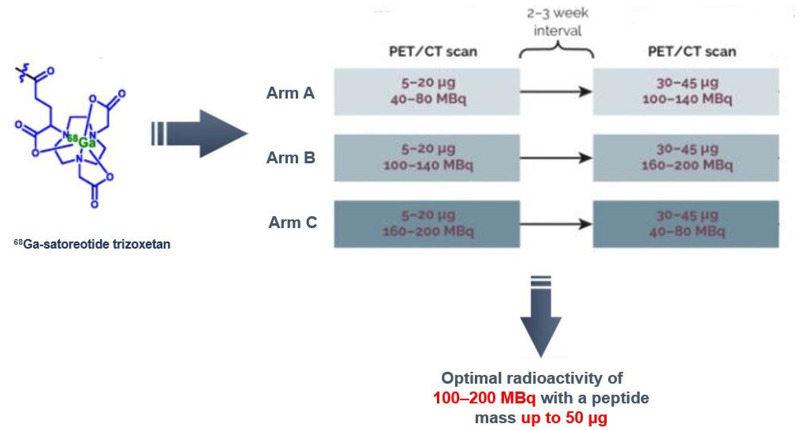

Gastroenteropancreatic neuroendocrine tumors (GEP-NETs) constitute a heterogeneous group of tumors, most of which overexpress somatostatin receptors (SSTRs) (*1*). The current standard for the diagnosis and staging of NETs is PET/CT using radiolabeled SSTR2 agonists such as ^68^Ga-DOTATATE, ^68^Ga-DOTATOC, or ^64^Cu-DOTATATE (*2*–*4*). The introduction of SSTR2 antagonists represents an important development in the field of NET imaging, as they bind to more receptors than SSTR2 agonists and therefore provide a higher tumor uptake, with better NET visualization (*5*–*8*).

^68^Ga-satoreotide trizoxetan (also known as ^68^Ga-IPN01070, ^68^Ga-NODAGA-JR11, or ^68^Ga-OPS202) is a new-generation somatostatin antagonist developed as a PET imaging agent for the detection and localization of NET lesions. It consists of the small somatostatin analog JR11 conjugated to the strong cyclical chelating agent 1,4,7-triazacyclononane,1-glutaric acid-4,7-acetic acid (NODAGA), which is radiolabeled with the isotope ^68^Ga. A previous prospective, single-center, open-label phase I/II imaging study (*9*), conducted on 12 patients with well-differentiated, low- or intermediate-grade, SSTR2-positive GEP-NETs, found that ^68^Ga-satoreotide trizoxetan, administered at a peptide mass ranging from 11 to 63 µg and an activity from 125 to 192 MBq, was associated with a significantly higher lesion-based overall sensitivity than was the SSTR2 agonist ^68^Ga-DOTATOC (88%–94% vs. 59%; *P* < 0.001). This observation was attributed mainly to the higher detection rate of metastases in the liver (*9*).

We report on a prospective, multicenter phase II trial designed to expand on the aforementioned phase I/II study (*9*) by confirming the optimal peptide mass and radioactivity ranges for ^68^Ga-satoreotide trizoxetan in patients with metastatic GEP-NETs. Furthermore, the design of this study was based on the U.S. Food and Drug Administration’s request for more data on the optimal diagnostic performance of ^68^Ga-satoreotide trizoxetan for PET imaging in a multicenter setting. It was hypothesized that an administered activity range of 40–80 MBq (1.08–2.16 mCi) would provide a reduced diagnostic signal compared with the recommended range of 100–200 MBq (2.70–5.41 mCi).

## MATERIALS AND METHODS

### Study Design

**FIGURE 1.**
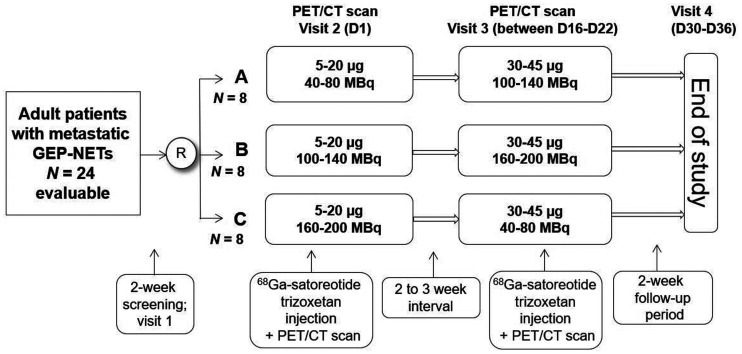
Study design. D = day; R = randomized.

This open-label, reader-masked, dose-confirmation, 2 × 3 factorial, randomized (1:1:1) phase II study (ClinicalTrials.gov identifier NCT03220217; EudraCT identifier 2016-004928-39) investigated 2 peptide mass ranges (5–20 and 30–45 µg) and 3 radioactivity ranges (40–80, 100–140, and 160–200 MBq) of ^68^Ga-satoreotide trizoxetan. The study was prospectively designed to enroll 8 patients in each of the 3 arms to ensure a balanced interdose evaluation. All patients received 2 doses of ^68^Ga-satoreotide trizoxetan on 2 separate visits, 2–3 wk apart, according to the randomization schedule of the 3 arms as shown in Figure 1.

Each patient underwent a total of 4 visits: a screening visit (visit 1) conducted within 2 wk before the first ^68^Ga-satoreotide trizoxetan administration; a visit on day 1 (visit 2) of the study, during which all patients received ^68^Ga-satoreotide trizoxetan at a peptide mass of 5–20 µg with 1 of the 3 ^68^Ga radioactivity ranges (40–80 MBq, arm A; 100–140 MBq, arm B; or 160–200 MBq, arm C); a visit on days 16–22 (visit 3), during which patients received the second dose of ^68^Ga-satoreotide trizoxetan at 30–45 µg and a radioactivity range different from that administered on day 1; and an end-of-study visit (visit 4) on days 30–36 for safety evaluation.

At screening, patient information was collected, including medical and surgical history; the primary tumor site, grade, and histopathology; and PET images demonstrating SSTR2-expressing lesions obtained within the previous 6 mo. A physical examination including vital sign assessment and laboratory tests (hematology, blood chemistry, and urinalysis) was performed at all study visits. A 12-lead electrocardiogram was recorded at screening and at the end of the study.

The study was conducted between September 2017 and October 2019 at 4 investigational sites in Austria, Denmark, and the United States. It was approved by all relevant ethics committees and conducted in accordance with the Declaration of Helsinki and the International Conference on Harmonisation guideline on good clinical practice. All patients provided written informed consent, and data were anonymized.

### Patients

Adults with pathologically confirmed, well-differentiated, functioning or nonfunctioning, metastatic grade 1/2 GEP-NETs were enrolled in this study. All patients had to have at least 2 but no more than 25 lesions per organ expressing SSTR2, which were identified with prior SSTR2 agonist PET scans in either the primary tumor site or in key organs (liver, lymph nodes, bones, and lungs). The limits were to ensure that a total number of lesions could be counted for statistical evaluation. Patient admissibility based on the number of SSTR2-expressing lesions was confirmed centrally by an independent nuclear medicine physician. Other inclusion criteria were an Eastern Cooperative Oncology Group performance status of 0–2, a body weight of 50–110 kg, and adequate hepatic, renal, and hematologic functions.

Key exclusion criteria were treatment with short- or long-acting somatostatin analogs within 24 h or 28 d, respectively, before either ^68^Ga-satoreotide trizoxetan injection, and any condition that might preclude the acquisition of high-quality PET or CT images.

### Imaging

At each of the 4 study centers, whole-body PET imaging was performed at both visit 2 and visit 3 of the study 50–70 min after intravenous injection of ^68^Ga-satoreotide trizoxetan, using Siemens Biograph dedicated PET/CT scanners, with an acquisition time of 2–4 min per bed position. All PET scans were acquired in list mode, including time-of-flight capability. Whole-body, low-dose CT images were acquired for localization and attenuation correction. During the same visits, patients also underwent contrast-enhanced CT (CECT), performed independently on a dedicated CT scanner and used as the standard of truth.

All images, including the prescreening SSTR agonist images, were sent to an imaging core lab (Keosys) after anonymization. After quality control assessment, the images were reviewed on a dedicated workstation. The PET images with and without the CT scans were evaluated by 2 experienced nuclear medicine physicians and 1 adjudicator for discordant cases. In parallel, CECT scans were read by 2 other radiologists, with a third adjudicating discordances. To minimize bias, the independent readers were unaware of the patient data, any information related to the study site, injected dose, and the temporal sequence of images.

### Radiopharmaceutical

^68^Ga-satoreotide trizoxetan was prepared at the study center’s local radiopharmacy, using the clinical trial dose kit provided by Beaufour Ipsen Industries, by a 2-step aseptic compounding process. This process included, first, reconstitution of sterile vial A containing the satoreotide trizoxetan precursor and excipients with 1 mL of solvent consisting of a solution of sterile sodium acetate from vial B and, second, radiolabeling of satoreotide trizoxetan by the addition of a 5-mL sterile hydrochloric acid solution of ^68^Ga, eluted from a sterile pharmaceutical-grade ^68^Ge/^68^Ga generator (Eckert and Ziegler Radiopharm).

The total amount of radioactivity injected by a slow-push intravenous injection into each patient was determined by measuring the radioactivity in the syringe before and after injection, using a standard dose calibrator. The peptide dose corresponded to injected volume (mL) × 8.33 µg/mL.

### Efficacy Assessments

All efficacy endpoints were assessed at the primary tumor site and in key organs (liver, lymph nodes, lungs, and bones) and were also evaluated in quartiles of radioactivity per baseline body weight expressed as MBq/kg. The primary endpoint of the study was the ratio of the number of lesions detected by ^68^Ga-satoreotide trizoxetan PET/CT and by PET alone to the number of lesions identified by CECT, for each of the 6 peptide mass and radioactivity range combinations. CECT was used as the standard of truth to provide a standardized denominator to make valid comparisons by peptide mass and radioactivity range.

Secondary efficacy endpoints included mean and median tumor-to-background ratios calculated by radioactivity range per patient’s body weight, preliminary diagnostic sensitivity of ^68^Ga-satoreotide trizoxetan PET/CT based on the number of lesions per patient compared with the standard of truth, and the absolute number of lesions detected by ^68^Ga-satoreotide trizoxetan PET/CT and the difference from the number of lesions detected by CECT. Preliminary diagnostic sensitivity was calculated as the number of lesions detected by ^68^Ga-satoreotide trizoxetan PET/CT and CECT divided by (number of lesions detected by ^68^Ga-satoreotide trizoxetan PET/CT and CECT scan plus number of lesions detected by CECT but not by ^68^Ga-satoreotide trizoxetan PET/CT).

### Safety Assessments

The safety and tolerability of ^68^Ga-satoreotide trizoxetan were assessed throughout the study on the basis of adverse events (AEs), which were graded according to the National Cancer Institute Common Terminology Criteria for Adverse Events (version 5.0) and coded using the Medical Dictionary for Regulatory Activities (version 22.1), laboratory results (hematologic, biochemical, and urologic), physical examinations, vital signs, and electrocardiography.

### Statistical Analysis

Statistical analysis was descriptive; consequently, no formal sample size calculation was performed. Continuous variables were presented as mean, SD, median, and range, whereas categoric variables were described by counts and percentages. The safety population was defined as all patients who received at least 1 dose of ^68^Ga-satoreotide trizoxetan. By contrast, to ensure a balanced and adequate assessment for each dose regimen, only the first 8 patients in each arm who successfully completed all ^68^Ga-satoreotide trizoxetan PET/CT scans were used in the efficacy analysis and were consequently included in the per-protocol population.

All statistical analyses were performed using SAS, version 9.4 (SAS Institute). Missing values were not replaced.

## RESULTS

### Patients

**FIGURE 2.**
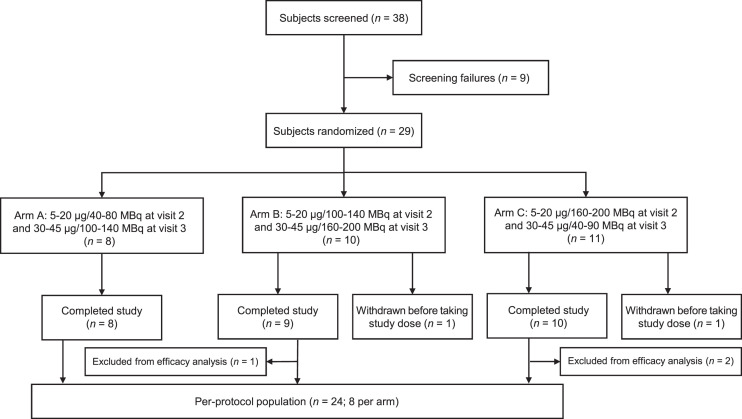
Patient disposition.

In total, 29 patients were enrolled in the study, with a median age of 63.0 y (Table 1). Two patients withdrew from the study before receiving ^68^Ga-satoreotide trizoxetan, leaving 27 patients in the safety population (8 in arm A, 9 in arm B, and 10 in arm C), as illustrated in Figure 2. The per-protocol population consisted of 24 patients (8 per arm) as initially planned.

**TABLE 1:** Baseline Demographic Characteristics in Randomized Population

Characteristic	Arm A (*n* = 8)	Arm B (*n* = 10)	Arm C (*n* = 11)	Overall (*n* = 29)
Age (y)				
Median	71.5	69.5	59.0	63.0
Range	54–84	60–78	36–78	36–84
Sex (*n*)				
Male	6 (75.0%)	4 (40.0%)	9 (81.8%)	19 (65.5%)
Female	2 (25.0%)	6 (60.0%)	2 (18.2%)	10 (34.5%)
Weight (kg)				
Median	81.0	85.3	86.0	83.0
Range	77–98	52–109	56–106	52–109
BMI (kg/m^2^)				
Median	26.4	28.8	26.3	26.4
Range	24–35	21–34	18–40	18–40
ECOG (*n*)				
0	7 (87.5%)	8 (80.0%)	9 (81.8%)	24 (82.8%)
1	1 (12.5%)	2 (20.0%)	2 (18.2%)	5 (17.2%)

BMI = body mass index; ECOG = Eastern Cooperative Oncology Group performance status.

Baseline demographic and disease characteristics were overall well balanced among the 3 study arms, with the small intestine being the most frequent primary-tumor site and liver and lymph nodes the most frequent metastasis locations (Tables 1 and 2). Overall, 28 of the 29 (96.6%) patients received at least 1 prior treatment, including somatostatin analogs in 26 (89.7%) patients, ^177^Lu-DOTATATE in 17 (58.6%), and everolimus in 3 (10.3%). There were no intercurrent treatments reported between radiolabeled SSTR agonists and antagonists or between 2 consecutive SSTR antagonists at any time during the study in any patient.

**TABLE 2:** Baseline Disease Characteristics in Per-Protocol Population

Characteristic	Arm A (*n* = 8)	Arm B (*n* = 8)	Arm C (*n* = 8)	Overall (*n* = 24)
Site of primary tumor				
Small intestine	6 (75.0)	4 (50.0)	6 (75.0)	16 (66.7)
Pancreas	1 (12.5)	1 (12.5)	1 (12.5)	3 (12.5)
Large intestine	1 (12.5)	3 (37.5)	1 (12.5)	5 (20.8)
Location of metastasis				
Liver	8 (100.0)	6 (75.0)	8 (100.0)	22 (91.7)
Lymph nodes	5 (62.5)	8 (100.0)	6 (75.0)	19 (79.2)
Bones	2 (25.0)	1 (12.5)	2 (25.0)	5 (20.8)
Lungs	2 (25.0)	0	2 (25.0)	4 (16.7)

Data are number followed by percentage in parentheses.

All 24 patients in the per-protocol population had a prior SSTR scan performed within a median of 1.6 mo (range, 0.1–6.0 mo) from screening: 14 (58.3%) had a PET/CT scan with ^68^Ga-DOTATOC, 9 (37.5%) with ^64^Cu-DOTATATE, and 1 (4.2%) with ^68^Ga-DOTATATE. The median total number of SSTR-positive lesions detected by prior SSTR agonist scans was 14.5 (range, 6.0–94.0). The median number of positive SSTR lesions was 1.0 (range, 0–1.0) in the primary tumor site, 9.5 (range, 0–37.0) in the liver, 5.0 (range, 0–36.0) in the lymph nodes, and 0.5 (range, 0–38.0) in the bones. Three patients who were enrolled had more than 30 lesions identified in the liver or in the lymph nodes.

### Efficacy

For all organs combined, the median number of lesions detected by ^68^Ga-satoreotide trizoxetan PET/CT or PET alone was at least twice as high as the number detected by CECT across the 6 peptide mass and radioactivity range combinations. This increase was reflected by a median relative lesion count, that is, the ratio of the number of lesions detected by ^68^Ga-satoreotide trizoxetan PET/CT or PET alone to the number of lesions detected by CECT, ranging from 2.1 to 3.9. The results for the primary efficacy endpoint are shown in Table 3. When comparing the 2 administered peptide mass ranges, as well as the 3 radioactivity ranges of ^68^Ga-satoreotide trizoxetan, we found no specific distribution pattern in the median relative lesion count for all organs combined. However, a trend toward a lower median relative lesion count in the liver was noted for the 40- to 80-MBq range, compared with the higher radioactivity ranges (Table 3). When counting the number of lesions detected by ^68^Ga-satoreotide trizoxetan imaging in each of the 3 study arms, we found exact agreement between the 2 readers for all patients in arm B (100%), whereas the interreader agreement rate was 87.5% in arm A and 75% in arm C.

**TABLE 3:** Relative Lesion Count in Per-Protocol Population (*n* = 24), by Peptide Mass and Radioactivity Ranges

	All organs*		Liver		Lymph nodes	
Parameter	PET/CT	PET	PET/CT	PET	PET/CT	PET
Peptide mass range						
5–20 µg	2.7 (0.64–16.25) (*n* = 24)	2.6 (0.73–19.00) (*n* = 24)	2.3 (0.73–9.00) (*n* = 17)	2.6 (0.67–7.00) (*n* = 17)	2.0 (0.00–8.00) (*n* = 13)	2.3 (0.50–10.00) (*n* = 13)
30–45 µg	2.7 (0.82–13.50) (*n* = 24)	2.8 (0.68–14.50) (*n* = 24)	3.0 (0.76–11.00) (*n* = 17)	2.8 (0.62–9.00) (*n* = 17)	1.3 (0.00–12.00) (*n* = 13)	2.0 (0.50–14.00) (*n* = 13)
Radioactivity range						
40–80 MBq	3.1 (0.73–15.00) (*n* = 16)	2.6 (0.68–19.00) (*n* = 16)	2.2 (0.73–9.00) (*n* = 13)	2.6 (0.62–6.00) (*n* = 13)	2.0 (1.00–3.00) (*n* = 7)	2.7 (0.75–4.00) (*n* = 7)
100–140 MBq	2.6 (0.64–13.50) (*n* = 16)	2.8 (1.00–14.50) (*n* = 16)	3.0 (0.83–8.00) (*n* = 9)	3.3 (0.67–7.00) (*n* = 9)	1.3 (0.00–8.00) (*n* = 9)	2.0 (0.50–10.00) (*n* = 9)
160–200 MBq	2.6 (0.82–16.25) (*n* = 16)	2.7 (0.91–13.50) (*n* = 16)	2.7 (0.86–11.00) (*n* = 12)	2.8 (0.86–9.00) (*n* = 12)	1.3 (0.00–12.00) (*n* = 10)	2.2 (0.50–14.00) (*n* = 10)

*Primary tumor site plus key organs (liver, lymph nodes, lungs, and bones).

Data are median and range. Relative lesion count is number of lesions detected by ^68^Ga-satoreotide trizoxetan PET/CT or PET alone divided by number of lesions detected by CECT. For liver and lymph nodes, number of analyzed patients were those who had ≥2 lesions detected by CECT on given organ.

Similarly, no pattern was found indicating a possible association between the radioactivity range per patient’s body weight (MBq/kg) and the relative lesion count or the absolute number of lesions detected by ^68^Ga-satoreotide trizoxetan (Table 4). There was also no observed association between the radioactivity range per patient weight and the tumor-to-background ratio (Table 5).

**TABLE 4:** Overall Relative Lesion Count in Per-Protocol Population (*n* = 24), by Radioactivity Range per Patient’s Baseline Body Weight

Parameter	0.69–0.97 MBq/kg (*n* = 14)	0.97–1.55 MBq/kg (*n* = 10)	1.55–2.09 MBq/kg (*n* = 13)	2.09–3.72 MBq/kg (*n* = 11)
Relative lesion count				
Mean ± SD	4.3 ± 4.04	2.9 ± 1.88	3.6 ± 3.31	4.0 ± 4.15
Median	3.5	2.5	2.1	2.6
Range	0.73–15.00	0.64–7.00	0.82–13.50	1.67–16.25
Absolute number of detected lesions				
Mean ± SD	26.9 ± 25.95	18.1 ± 20.62	25.9 ± 21.45	26.8 ± 18.59
Median	16.5	11.5	20.0	19.0
Range	7–94	6–75	8–73	10–65

Overall relative lesion count is number of lesions detected by ^68^Ga-satoreotide trizoxetan in all organs divided by number of lesions detected by CECT. Absolute number of detected lesions corresponds here to absolute number of lesions detected by ^68^Ga-satoreotide trizoxetan in all organs.

**TABLE 5:** Tumor-to-Background Ratio for Liver and Lymph Nodes in Per-Protocol Population (*n* = 24), by Radioactivity Range per Patient’s Baseline Body Weight

Organ	Parameter	0.69–0.97 MBq/kg (*n* = 14)	0.97–1.55 MBq/kg (*n* = 10)	1.55–2.09 MBq/kg (*n* = 13)	2.09–3.72 MBq/kg (*n* = 11)
Liver	n*	11	6	9	8
	Mean ± SD	7.1 ± 5.90	4.3 ± 1.03	4.9 ± 2.95	6.2 ± 3.68
	Median	4.3	4.0	4.1	5.1
	Range	3.07–22.48	3.35–6.13	2.13–10.75	2.57–12.63
Lymph nodes	n*	5	6	7	8
	Mean ± SD	10.1 ± 7.25	8.3 ± 5.18	9.2 ± 5.16	5.1 ± 3.17
	Median	7.4	5.3	11.4	4.5
	Range	3.53–18.69	4.47–16.10	2.87–15.00	1.54–11.65

*Number of patients with lesions in either liver or lymph nodes. Number of patients with lesions in primary tumor site and bones was too small (≤3 in each category) to allow meaningful interpretation.

The preliminary diagnostic sensitivity of ^68^Ga-satoreotide trizoxetan PET/CT, using CECT as the standard of truth, ranged from a median of 85% to 87% across different peptide mass and radioactivity ranges (Table 6).

**TABLE 6:** Preliminary Diagnostic Sensitivity of ^68^Ga-Satoreotide Trizoxetan PET/CT in Per-Protocol Population (*n* = 24), by Peptide Mass and Radioactivity Range

	Peptide mass range		Radioactivity range				
Parameter	5–20 μg (*n* = 24)	30–45 μg (*n* = 24)	40–80 MBq (*n* = 16)	100–140 MBq (*n* = 16)	160–200 MBq (*n* = 16)	100–200 MBq (*n* = 24)	40–200 MBq (*n* = 24)
Mean ± SD	79 ± 24	78 ± 28	86 ± 14	71 ± 34	77 ± 25	75 ± 29	80 ± 24
Median	85	87	87	85	86	85	85
Range	8–100	0–100	64–100	0–100	8–100	0–100	8–100

Diagnostic sensitivity is number of lesions detected by ^68^Ga-satoreotide trizoxetan PET/CT and by CECT divided by (number of lesions detected by ^68^Ga-satoreotide trizoxetan PET/CT and by CECT plus number of lesions detected by CECT but not ^68^Ga satoreotide trizoxetan PET/CT) × 100.

### Safety

All patients included in the safety population (*n* = 27) received 2 injections of ^68^Ga-satoreotide trizoxetan during the study. In total, 33 AEs were reported in 18 patients (66.7%), classified as grade 1 (23 AEs in 15 patients), grade 2 (9 in 5), or grade 3 (1 in 1 [hypertriglyceridemia]). Overall, 14 drug-related AEs were reported in 7 patients (25.9%), all grade 1 or 2, which included injection site pain (4 events), nausea (2 events), proteinuria (2 events), feeling cold (2 events), flushing (1 event), alopecia (1 event), diarrhea (1 event), and fatigue (1 event). Drug-related AEs occurred 1–2 d after the last dose of ^68^Ga-satoreotide and were resolved within 15 d. All but one of the patients completely recovered from all drug-related AEs; the patient who did not completely recover reported alopecia, which subsequently resolved, with sequelae. No serious AEs and no postdose AEs leading to withdrawal or death were reported.

## DISCUSSION

This multicenter, randomized, factorial phase II study evaluated the optimal dose range of ^68^Ga-satoreotide trizoxetan. The results showed that the ratio of the number of lesions detected by ^68^Ga-satoreotide trizoxetan to the number of lesions detected by CECT was overall consistent across different peptide mass and radioactivity ranges, with no dependence on subject weight; however, a lower relative lesion count (median) in the liver was noted for the 40- to 80-MBq range than for the higher radioactivity ranges. As anticipated with radiolabeled somatostatin analogs, the number of lesions identified by ^68^Ga-satoreotide trizoxetan was consistently higher than that identified by CECT in organs where lesions were present.

For the routine clinical setting, PET/CT images should be considered the primary method of evaluation in NET patients (*10*,*11*). The primary efficacy analysis found that, with ^68^Ga-satoreotide trizoxetan PET/CT scans (and also PET alone), there were no noticeable differences between the 2 peptide mass ranges in the number of identified lesions for any organs, with a median relative lesion count of 2.7 for both peptide mass ranges. Hence, on the basis of the results of primary and secondary endpoints, ^68^Ga-satoreotide trizoxetan imaging was not peptide mass–dependent. This finding is corroborated by a phase I/II imaging study by Nicolas et al. (*9*) that found no significant differences between 2 administered peptide mass ranges (14 ± 4 and 50 ± 15 µg) in the number of malignant liver or lymph node lesions detected per patient or the tumor-to-background ratios, indicating a high reproducibility for ^68^Ga-satoreotide trizoxetan PET/CT, regardless of the administered amount of peptide. Thereby, the present study, along with the findings of the Nicolas et al. study (*9*), confirms that the optimal peptide mass of ^68^Ga-satoreotide trizoxetan for diagnostic imaging of GEP-NETs can be up to 50 µg, which is congruent with the current European Association of Nuclear Medicine administration guidelines for ^68^Ga-labeled SSTR2 agonists (*10*).

Regarding the optimal administered radioactivity of ^68^Ga-satoreotide trizoxetan, the present study confirms that ranges of 100–140 and 160–200 MBq provide optimal imaging results. By contrast, the 40- to 80-MBq radioactivity range is associated with a trend toward a lower median relative lesion count in the liver, the predominant site of metastases in patients with GEP-NETs (*12*); therefore, further development will not be pursued for this radioactivity range, which, of note, was not tested in prior studies. The 40- to 80-MBq radioactivity range might also be associated with a reduced ratio of receptor-bound tracer to free tracer, resulting in declined image contrast and poor detection of GEP-NETs (*13*,*14*).

The absence of notable diagnostic performance and safety differences between the radioactivity ranges of 100–140 and 160–200 MBq, when discounting the 40- to 80-MBq range, confirms the optimal radioactivity range and ascertains the 100- to 200-MBq range as the appropriate activity for future use of ^68^Ga-satoreotide trizoxetan. This result is in keeping with the European Association of Nuclear Medicine guidelines, which recommend an administered radioactivity range of between 100 and 200 MBq for ^68^Ga-labeled SSTR2 agonists, depending on the technical characteristics of the PET scanner and the patient’s body weight (*10*). Similarly, the Society of Nuclear Medicine and Molecular Imaging recommends administration of ^68^Ga-labeled SSTR2 agonists at a radioactivity of between 111 and 259 MBq, while taking into account the patient’s body weight (*11*). Although there is a possibility of narrowing the radioactivity window of ^68^Ga-satoreotide trizoxetan from 100–200 to 100–140 MBq, adopting the wider, guideline-recommended radioactivity range of 100–200 MBq offers increased flexibility and feasibility in routine clinical practice while maintaining dosing similarity to other ^68^Ga-labeled products. The absence of a clear dose–response relationship in the present study might be related to factors such as receptor density heterogeneity, hypoxia, interstitial pressure, necrosis, and tumor heterogeneity (*15*).

Of significant note, this study did not find a weight-dependent effect of ^68^Ga-satoreotide trizoxetan across the evaluated quartiles of radioactivity per body weight. This lack of a weight-dependent effect ideally provides an opportunity for an activity range of ^68^Ga-satoreotide trizoxetan (100–200 MBq) to be prescribed, regardless of body weight. By contrast, ^68^Ga-labeled SSTR2 agonists require dosing per body mass (*10*,*11*,*16*). In a recent prospective study from The Netherlands conducted among 21 patients with NETs who underwent whole-body ^68^Ga-DOTATATE PET/CT, Cox et al. reported that, of all patient-dependent parameters, body mass showed the strongest correlation (coefficient of determination, 0.60) with normalized signal-to-noise ratio (*16*). Importantly, the absence of a weight-dependent effect represents a practical advantage for ^68^Ga-satoreotide trizoxetan, as an administered activity within a predetermined range instead of a weight-based administered activity not only is more convenient but also reduces the possibility of dosing errors.

The frequency and nature of the AEs reported with ^68^Ga-satoreotide trizoxetan in the present study did not raise any safety concern. Many of these clinical manifestations (e.g., nausea, flushing, diarrhea, and fatigue) are common in patients with NETs. This safety profile compares favorably with that in the Nicolas et al. imaging study (*17*), in which no severe AEs or postdose AEs leading to withdrawal or death were reported.

This study was limited mainly by a small sample size, which negated the use of a formal statistical analysis; thus, descriptive statistical analyses were applied. In addition, although not affecting image readability, the enrollment of 3 patients with more than 25 lesions per organ did increase lesion burden and skewed distributions in some instances. The study was also designed to evaluate 5 different organs (primary tumor site, liver, lymph nodes, lungs, and bones). However, because few lesions were identified in certain organs, meaningful results on the number of identified lesions were available only for all organs combined, the liver, and lymph nodes. Nevertheless, these limitations were balanced by a robust study design that allowed inter- and intraindividual comparisons across different peptide mass and radioactivity range combinations, which were evaluated at patient and lesion levels for both ^68^Ga-satoreotide trizoxetan PET/CT and PET alone.

## CONCLUSION

The overall results of this study confirm that the optimal administered peptide mass of ^68^Ga-satoreotide trizoxetan is up to 50 µg with a radioactivity of 100–200 MBq, as is in line with current guidelines for administration of ^68^Ga-labeled radiopharmaceuticals (*10*,*11*). This phase II study also confirmed that the overall safety profile of ^68^Ga-satoreotide trizoxetan is acceptable for continued clinical development.

## DISCLOSURE

This study was funded by Ipsen (Boulogne, France). Partner 4 Health (Paris, France) provided medical writing support (sponsored by Ipsen) in accordance with Good Publication Practice (GPP3) guidelines. Irene Virgolini has acted as a consultant for Ipsen and Advanced Accelerator Applications. Henning Grønbæk serves on the advisory board of Ipsen. Colin Miller has acted as a consultant for Ipsen, CytoSite Bio, and Alacrita and is a managing partner at the Bracken Group. Thomas Rohban is a managing partner at Partner 4 Health and has acted as a consultant for Ipsen. Sandy McEwan and Christine Powell are employees at Ipsen Bioscience. No other potential conflict of interest relevant to this article was reported.

## Notes

KEY POINTS **QUESTION:** What are the optimal peptide mass and radioactivity ranges for ^68^Ga-satoreotide trizoxetan administered as a PET imaging agent in patients with GEP-NETs? **PERTINENT FINDINGS:** In this prospective, randomized, open-label, factorial-design phase II study, the ratio of the number of lesions detected by ^68^Ga-satoreotide trizoxetan to the number detected by CECT was overall consistent across different peptide mass and radioactivity ranges, with no weight-dependent effect. However, a radioactivity range of 40–80 MBq was associated with a trend toward a lower median relative lesion count in the liver, the predominant site of metastasis in patients with GEP-NETs. **IMPLICATIONS FOR PATIENT CARE:** A radioactivity of 100–200 MBq with a peptide mass of up to 50 µg was confirmed as the optimal dosing regimen for ^68^Ga-satoreotide trizoxetan to be used in future phase III clinical trials.
